# Inhibition of β-Catenin/CREB Binding Protein Signaling Attenuates House Dust Mite-Induced Goblet Cell Metaplasia in Mice

**DOI:** 10.3389/fphys.2021.690531

**Published:** 2021-07-27

**Authors:** Virinchi N. S. Kuchibhotla, Malcolm R. Starkey, Andrew T. Reid, Irene H. Heijink, Martijn C. Nawijn, Philip M. Hansbro, Darryl A. Knight

**Affiliations:** ^1^Priority Research Centre for Healthy Lungs, Hunter Medical Research Institute, New Lambton Heights, NSW, Australia; ^2^School of Biomedical Sciences and Pharmacy, The University of Newcastle, Callaghan, NSW, Australia; ^3^GRIAC Research Institute, University Medical Center Groningen, Groningen, Netherlands; ^4^Department of Pathology and Medical Biology, Laboratory of Experimental Pulmonology and Inflammation Research, University of Groningen, Groningen, Netherlands; ^5^Priority Research Centre GrowUpWell and Hunter Medical Research Institute, Faculty of Health and Medicine, The University of Newcastle, Newcastle, NSW, Australia; ^6^Department of Immunology and Pathology, Central Clinical School, Monash University, Melbourne, VIC, Australia; ^7^School of Medicine and Public Health, The University of Newcastle, Callaghan, NSW, Australia; ^8^Department of Pulmonology, University Medical Center Groningen, University of Groningen, Groningen, Netherlands; ^9^Centre for Inflammation, Centenary Institute, Sydney, NSW, Australia; ^10^School of Life Sciences, Faculty of Science, University of Technology Sydney, Sydney, NSW, Australia; ^11^Providence Health Care Research Institute, Vancouver, BC, Canada; ^12^Department of Anesthesiology, Pharmacology and Therapeutics, The University of British Columbia, Vancouver, BC, Canada

**Keywords:** asthma, airway inflammation, β-catenin, ICG-001, goblet cell metaplasia

## Abstract

Excessive mucus production is a major feature of allergic asthma. Disruption of epithelial junctions by allergens such as house dust mite (HDM) results in the activation of β-catenin signaling, which has been reported to stimulate goblet cell differentiation. β-catenin interacts with various co-activators including CREB binding protein (CBP) and p300, thereby regulating the expression of genes involved in cell proliferation and differentiation, respectively. We specifically investigated the role of the β-catenin/CBP signaling pathway in goblet cell metaplasia in a HDM-induced allergic airway disease model in mice using ICG-001, a small molecule inhibitor that blocks the binding of CBP to β-catenin. Female 6- 8-week-old BALB/c mice were sensitized to HDM/saline on days 0, 1, and 2, followed by intranasal challenge with HDM/saline with or without subcutaneous ICG-001/vehicle treatment from days 14 to 17, and samples harvested 24 h after the last challenge/treatment. Differential inflammatory cells in bronchoalveolar lavage (BAL) fluid were enumerated. Alcian blue (AB)/Periodic acid–Schiff (PAS) staining was used to identify goblet cells/mucus production, and airway hyperresponsiveness (AHR) was assessed using invasive plethysmography. Exposure to HDM induced airway inflammation, goblet cell metaplasia and increased AHR, with increased airway resistance in response to the non-specific spasmogen methacholine. Inhibition of the β-catenin/CBP pathway using treatment with ICG-001 significantly attenuated the HDM-induced goblet cell metaplasia and infiltration of macrophages, but had no effect on eosinophils, neutrophils, lymphocytes or AHR. Increased β-catenin/CBP signaling may promote HDM-induced goblet cell metaplasia in mice.

## Introduction

Asthma is broadly characterized by chronic inflammation and remodeling of the airways, excessive airway mucus production, reversible airflow obstruction with loss of lung function (Fehrenbach et al., 2017). The airway epithelium is the first line of defense against inhaled allergens and is more susceptible to damage by allergens such as house dust mite (HDM) in asthma. This results in the deterioration of cellular junctions (Heijink et al., 2014), and release of pro-inflammatory cytokines like Chemokine (C-C motif) ligand 20 (CCL20), CCL17, interleukin (IL)-25, IL-33 and thymic stromal lymphopoietin (TSLP), which activate and attract immune cells like dendritic cells (DCs) and macrophages, promoting the differentiation of type-2 cells (Heijink et al., 2007; Hansbro et al., 2013; Hallstrand et al., 2014). T helper 2 (Th2) cells and innate lymphoid cells (ILCs) release various cytokines including IL-4, IL-5, and IL-13, which activate B-lymphocytes, induce the infiltration of eosinophils, airway hyperresponsiveness (AHR) (Thorburn et al., 2012; Liu et al., 2017), and enhance the differentiation of goblet cells (Hammad and Lambrecht, 2015; Reid et al., 2018; Starkey et al., 2019). Goblet cells are secretory cells that produce mucus, which consists of polypeptides, water, DNA, enzymes and high molecular weight glycoproteins called mucins (Williams et al., 2006). MUC5AC and MUC5B are the major mucins secreted by goblet cells, and their expression is increased in asthma and is associated with goblet cell metaplasia (Bonser and Erle, 2017; Vieira Braga et al., 2019). MUC5AC production is regulated by the transcription factors SAM pointed domain containing ETS transcription factor (SPDEF) and forkhead box a2 (FOXA2), which activate and repress the expression of MUC5AC, respectively (Evans et al., 2009; Bonser and Erle, 2017).

**FIGURE 1:**
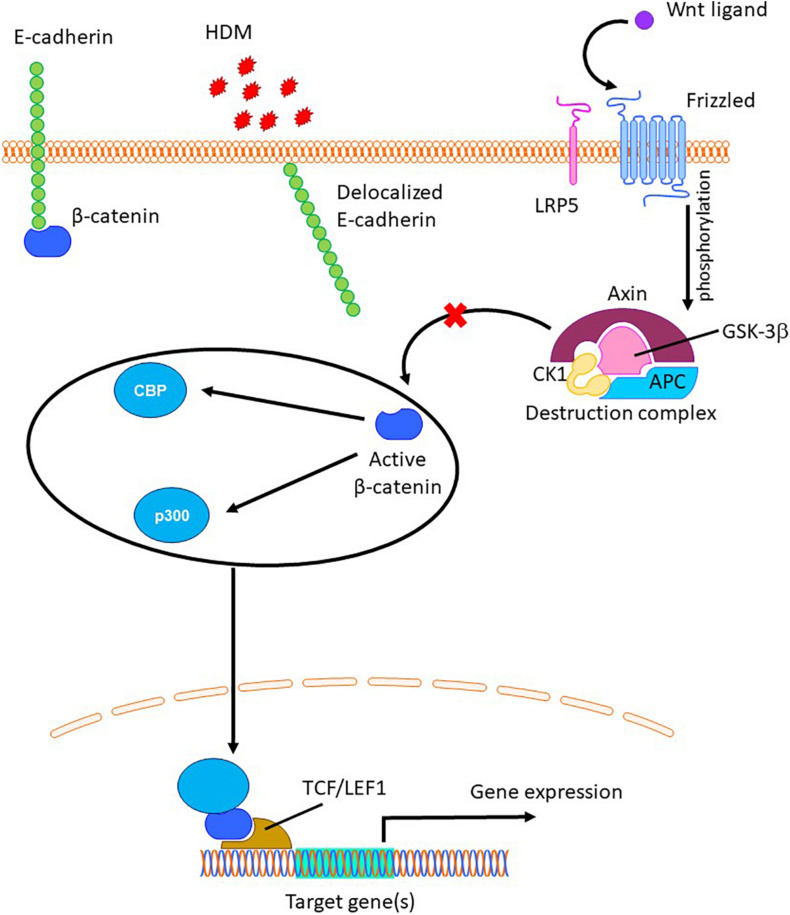
The role of β-catenin in regulation of gene expression. Allergens like house dust mite (HDM) can delocalize junctional E-cadherin (Post et al., 2012), resulting in the release of β-catenin into cytoplasm, where is targeted for proteolytic degradation by the destruction complex (Valenta et al., 2012). Upon Wnt ligand mediated phosphorylation and inactivation of GSK-3β, cytosolic β-catenin escapes from the destruction complex, translocates to the nucleus and binds to TCF/LEF group of transcriptional factors and other co-activators such as CBP and p300, resulting transcription of target genes (Valenta et al., 2012).

Adherens junctions, which mainly constitute the transmembrane protein E-cadherin, help in maintaining the integrity and function of the airway epithelium. Reduced expression of E-cadherin has been observed in airway epithelial cells from asthmatic donors along with decreased epithelial barrier function (Post et al., 2013). We have previously shown that allergens like HDM disrupt E-cadherin at the cell junctions and induce goblet cell metaplasia in mice (Post et al., 2012). In mice, embryonic knock-out of E-cadherin in airway epithelial cells induced club cell hyperplasia (Ceteci et al., 2012) and excessive mucus production (Post et al., 2018). The loss of E-cadherin leads to the delocalization of its intracellular binding partner β-catenin into the cytoplasm, where its levels are regulated by a destruction complex consisting of axin, adenomatous polyposis coli APC and GSK-3β. Activation of Wnt and/or growth factor signaling prevents phosphorylation and degradation of β-catenin by the destruction complex in the cytoplasm. The non-phosphorylated β-catenin, which is the transcriptionally active form of β-catenin, translocates to the nucleus and acts as a transcription factor to regulate the expression of various genes involved in cellular development and regulate cell fate decisions (Valenta et al., 2012; Figure 1). Increased transcriptional activity of β-catenin has been shown to induce goblet cell metaplasia in mice (Mucenski et al., 2005). The regulation of a variety of genes by β-catenin is possible because of its ability to bind to various transcriptional coactivators including CREB binding protein (CBP) and p300 leading to divergent cellular processes of cell migration and proliferation versus differentiation, respectively (Ma et al., 2005). Specifically, β-catenin/CBP signaling has been shown to regulate epithelial-to-mesenchymal transition of primary airway epithelial cells, leading to loss of epithelial markers such as E-cadherin (Nawijn et al., 2011; Moheimani et al., 2015). The small molecule inhibitor ICG-001 has been previously shown to inhibit β-catenin/CBP signaling by specifically binding to CBP, thereby preventing β-catenin from interacting with CBP (Emami et al., 2004). In addition, inhibition of β-catenin/CBP pathway by the small molecule inhibitor ICG-001 improved airway epithelial barrier function *in vitro* upon Ca^+2^ signaling-induced damage by stabilizing E-cadherin at cell junctions (Kuchibhotla et al., 2020). We hypothesized that inhibition of the β-catenin/CBP pathway upon HDM-induced E-cadherin loss using ICG-001 would attenuate HDM-induced goblet cell metaplasia and AHR in a mouse model of allergic asthma.

## Materials and Methods

### Mice

6–8 week old female BALB/c mice were obtained from the University of Newcastle’s central animal house and housed at the Hunter Medical Research Institute animal facility in individually ventilated cages in an SPF PC2 facility. Mice were provided standard rodent chow *ad libitum*. All protocols were approved by the University of Newcastle Animal Care and Ethics Committee.

### House Dust Mite Model of Allergic Airway Disease

**FIGURE 2:**
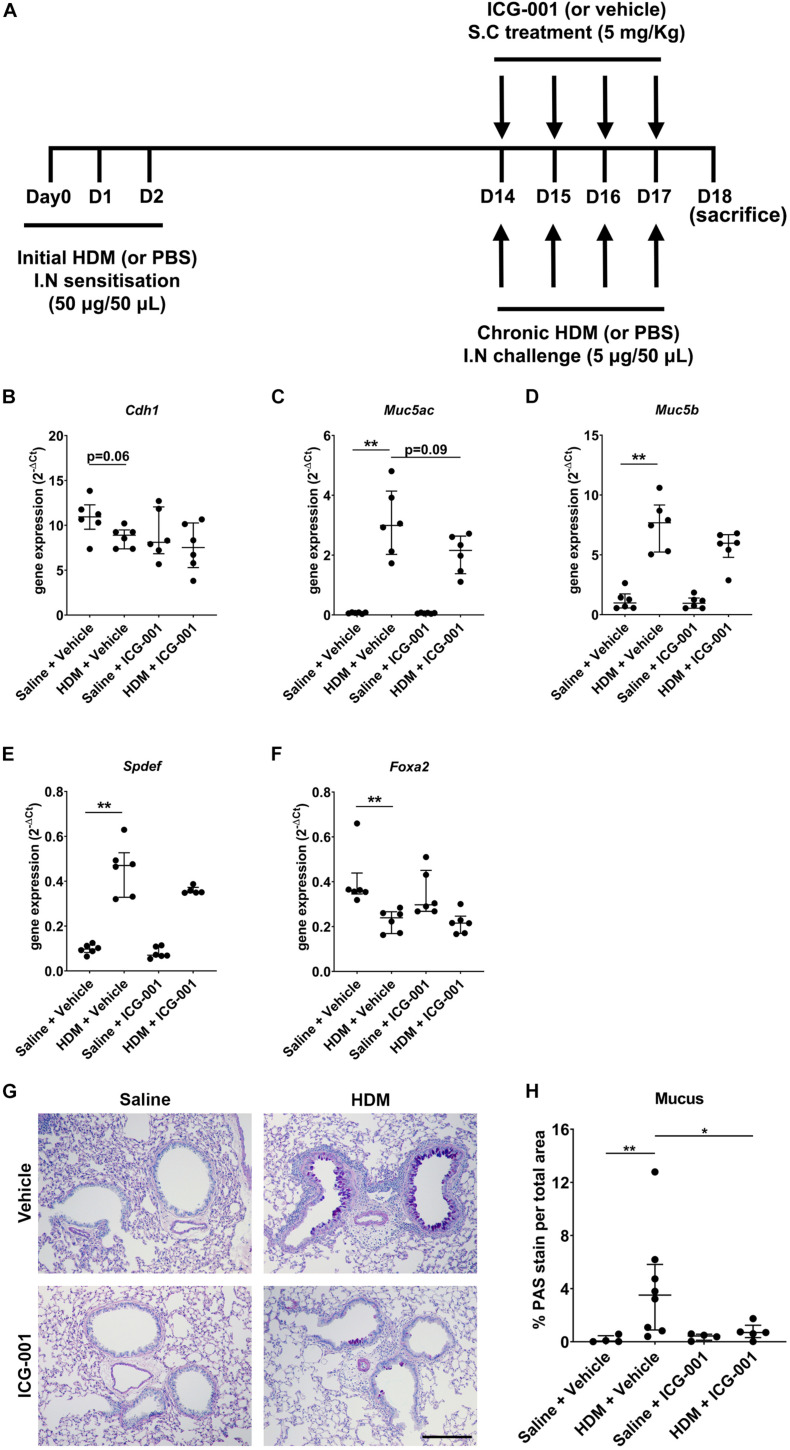
ICG-001 decreases HDM-induced goblet cell metaplasia. **(A)** HDM-induced allergic airway disease model in mice treated with the ICG-001 inhibitor. **(B–F)** Gene expression of *Cdh1* (*n* = 6), *Muc5ac* (*n* = 6), *Muc5b* (*n* = 6), *Spdef* (*n* = 5–6) and *Foxa2* (*n* = 6) normalized to *Hprt* (*n* = 6) **(G)** Alcian Blue (AB)/Periodic acid–Schiff (PAS) staining of lung sections from mice for mucus (*n* = 4–8), scale = 100 μm. **(H)** Semi-quantification of AB/PAS staining for mucus using color deconvolution (*n* = 4–8). Data is presented as median ± IQR; *p < 0.05, **p < 0.01, Mann–Whitney *U* test.

Mice (*n* = 6−8/group) were sensitized to HDM (*Dermatophagoides pteronyssinus*) extract [intranasal (i.n.): days 0, 1, and 2; 50 μg; Greer Labs, NC, United States] in sterile saline (50 μl) and challenged with HDM (i.n.: day 14–17; 5 μg in 50 μl saline) with/without ICG-001 (subcutaneous: day 14–17; 5 mg/Kg) under isoflurane anesthesia and euthanized on day 18 (Figure 2A) as previously described (Thorburn et al., 2012, 2015).

### Quantitative Polymerase Chain Reaction

Total RNA was extracted from lung homogenates using TRIZOL (Sigma, MO, United States) as previously described (Horvat et al., 2007; Essilfie et al., 2011; Kim et al., 2017b). Synthesis of cDNA was performed using high-capacity cDNA reverse transcription kit with RNase inhibitor (Thermo Fisher Scientific, Waltham, MA, United States) and T100 Thermal Cycler (Bio-Rad Laboratories, CA, United States). Quantitative real time PCR (qRT-PCR) targeting *Cdh1* (Mm01247357_m1, Thermo Fisher Scientific), *Muc5ac* (Mm01276718_m1, Thermo Fisher Scientific), *Muc5b* (Mm00466391_m1, Thermo Fisher Scientific), *Spdef* (Hs00171942_m1, Thermo Fisher Scientific), and *Foxa2* (Mm01976556_s1, Thermo Fisher Scientific) was performed on individual biological replicates, which were normalized to *Hprt* (Mm03024075_m1, Thermo Fisher Scientific) and presented as gene expression (2^–ΔCt^) as previously described (Schmittgen and Livak, 2008).

### Histological Analysis

Lung sections of 5 μm thickness were obtained for all the experimental mice and stained for goblet cells/mucins using standard Alcian blue (AB) pH 2.5 followed by periodic acid and Schiff’s reagent (PAS) as previously described (Horvat et al., 2007; Starkey et al., 2013; Reid et al., 2020). A minimum of eight different airways per section were imaged at 10× magnification and quantified using color deconvolution algorithm in ImageJ (National institute of Health) by one person as previously described (Horvat et al., 2007; Starkey et al., 2013; Reid et al., 2020). All the AB/PAS staining images were blinded and imported into ImageJ. The airway epithelial cells were selected by drawing a region of interest (ROI) and the outside area was deleted. The “Threshold color” was adjusted to select all airway epithelial cells and the total area of the airway epithelial cells was measured. Next, by selecting the “color deconvolution” function with H-PAS vector, the image was split into three different color channels, of which, the blue/magenta color channel was selected. The AB/PAS staining intensity was quantified using the “Threshold” function. The same threshold value is used all the images for consistency and eliminate any human bias. All the values of AB/PAS staining intensities and area of airway epithelial cells from different airways of a single image section were separately added to get the total AB/PAS staining intensity and total area of airway epithelial cells, respectively. Finally, the % AB/PAS staining was calculated using the following equation: (total AB/PAS staining intensity/total area of airway epithelial cells) × 100. Each data point represents the % AB/PAS staining of mucins per total area of the airway epithelial cells in a section from a single mouse.

### Immune Cell Quantification

Bronchoalveolar lavage fluid (2 ml) was prepared, and total cell numbers were determined with a hemocytometer. Cells prepared by cytocentrifugation (Shandon Cytospin; Thermo Fisher Scientific, Waltham, MA, United States) were stained with May-Grünwald-Giemsa and leukocytes were enumerated on the basis of morphologic criteria [200 cells by light microscopy (×40)] as previously described (Horvat et al., 2007; Kaiko et al., 2008; Thorburn et al., 2010). Further, eosinophils, neutrophils, lymphocytes, and macrophages were calculated and represented as the percentage of the total leukocytes in BAL fluid.

### Airway Hyperresponsiveness

Airway hyperresponsiveness was measured using plethysmography, which provides similar results as Flexivent in HDM models of experimental asthma (Horvat et al., 2007; Kim et al., 2017b,c). Mice were anesthetized [ketamine and xylazine (80–100 and 10 mg/kg, respectively); Troy Laboratories, Smithfield, NSW, Australia] and the tracheas were cannulated. Each cannula was connected to an inline aerosol administrator and ventilator, which were attached to a preamplifier and computer (Buxco, Wilmington, NC, United States) to analyze pressure and flow waveforms and to determine airway resistance and dynamic compliance. Mice were nebulized with saline followed by increasing doses of methacholine (Sigma) as previously described (Horvat et al., 2007; Kim et al., 2017b,c).

### Statistical Analysis

**FIGURE 3:**
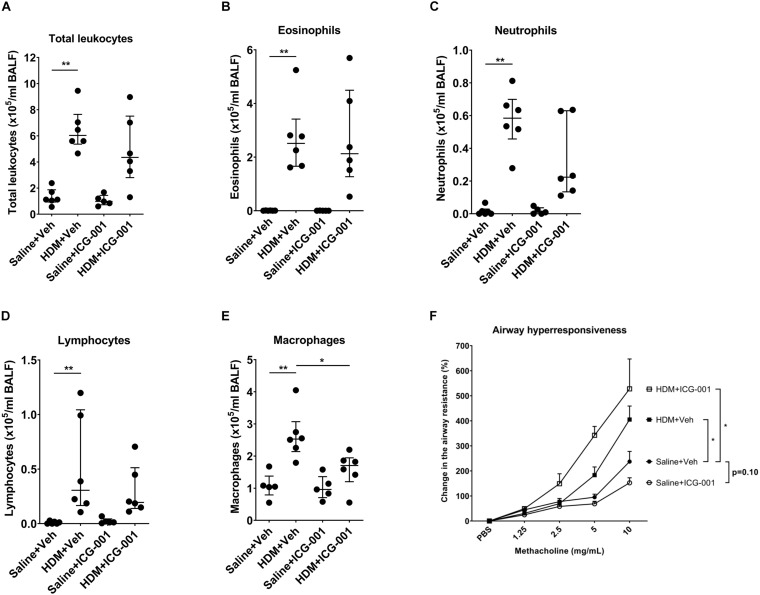
ICG-001 inhibits HDM-induced infiltration of macrophages but does not influence HDM-induced AHR. Numbers of **(A)** total leukocytes (*n* = 5–6), **(B)** eosinophils (*n* = 5–6), **(C)** neutrophils (*n* = 5–6), **(D)** lymphocytes (*n* = 5–6), and **(E)** macrophages (*n* = 5–6) present in BAL fluid of mice. Data is presented as median ± IQR; *n* = 5–6, *p < 0.05, **p < 0.01, Mann–Whitney *U* test. **(F)** Change in the methacholine-induced airway resistance (%) in mice following exposure to HDM and treated with ICG-001 or vehicle-treated controls. Data is presented as median ± IQR; *n* = 6, *p < 0.05, two-way ANOVA.

All statistical analyses were performed using GraphPad Prism (Graphpad software, San Diego, CA, United States). Non-parametric Mann–Whitney *U* test was performed to assess for significant differences in gene expression, staining quantification, and the infiltration of immune cells between different groups. For AHR, two-way analysis of variance (ANOVA) was used to compare different groups and multiple comparisons were done by uncorrected Dunn’s test. Outliers were identified by Grubb’s test. We detected one outlier in the HDM + ICG-001 group for the *Spdef* gene expression analysis (Figure 2F) and one outlier in the HDM + ICG-001 group for the AB/PAS staining quantification (Figure 2H) and removed these from the specific analyses. In addition, we also detected and removed one outlier each in the Saline + ICG-001 group for the measurement of total leukocytes, eosinophils, neutrophils, lymphocytes, and macrophages (Figures 3A–E and Supplementary Figures 1A–E), which are from a single mouse. *P* < 0.05 was considered statistically significant.

## Results

### ICG-001 Decreases HDM-Induced Goblet Cell Metaplasia *in vivo*

Sensitization of mice for 3 days from day 0 to 3 followed by exposure to HDM for 4 days from day 14 to 17 showed a strong trend (*p* = 0.06) toward a decrease in the mRNA expression of E-cadherin (*Cdh1*) compared to PBS-sensitized and PBS-challenged mice. In mice treated with ICG-001, no differences were observed in the *Cdh1* mRNA expression levels after HDM exposure compared to the PBS-exposed controls (Figure 2B). HDM significantly increased the mRNA expression of secretory mucins *Muc5ac* and *Muc5b* (Figures 2C,D). Treatment of mice with ICG-001 resulted in a trend (*p* = 0.09) toward the suppression of HDM-induced mRNA expression of *Muc5ac* but had no effect on *Muc5b* expression (Figures 2C,D). The *Muc5ac* transcriptional activator *Spdef* was significantly increased (Figure 2E) and the *Muc5ac* transcriptional repressor *Foxa2* was significantly decreased (Figure 2F) following HDM exposure, which was not affected by ICG-001 treatment (Figures 2E,F). The increase in *Muc5ac* and *Muc5b* expression was validated at the protein level by AB/PAS staining, revealing that HDM exposure promoted goblet cell metaplasia and mucus production compared to the PBS + Vehicle treated controls. Notably, HDM-induced goblet cell metaplasia and mucus production was significantly repressed by ICG-001 treatment (Figures 2G,H).

### ICG-001 Inhibits HDM-Induced Infiltration of Macrophages, but Does Not Influence HDM-Induced AHR

Airway inflammation with increases in leukocytes and notably eosinophils is a hallmark of the HDM-induced asthma model in mice. HDM significantly increased the total leukocyte counts. The majority of leukocytes in the BAL fluid of PBS exposed mice were macrophages (98.3 ± 1.6%) and the remaining fraction included eosinophils, neutrophils and lymphocytes (Supplementary Figure 1). Exposure to HDM significantly increased the number of eosinophils, neutrophils, lymphocytes, and to a lesser extent, macrophages in BAL fluid (Figures 3A–E), resulting in a decrease in the percentage of macrophages (Supplementary Figure 1E). ICG-001 did not have any effect on HDM-induced increases in the absolute numbers of total leukocytes including eosinophils, neutrophils, and lymphocytes (Figures 3A–D). ICG-001 treatment did result in a significant reduction in HDM-induced macrophages (Figure 3E). Methacholine induced a dose-dependent increase in airway resistance, which was significantly elevated by HDM sensitization and challenge (Figure 3F). In line with the lack of effect on inflammation, ICG-001 treatment did not suppress HDM-induced increase in the airway resistance (Figure 3F).

## Discussion

In this study, we investigated the role of β-catenin/CBP signaling in allergen-induced manifestations of asthma. HDM exposure reduced the expression of E-cadherin, which was accompanied by increases in *Muc5ac*, *Muc5b*, and *Spdef* expression, decreased *Foxa2* expression, the induction of goblet cell metaplasia and the infiltration of inflammatory cells, thereby representing the phenotype of asthmatic airway epithelium. Specific inhibition of β-catenin/CBP signaling using ICG-001 significantly decreased goblet cell metaplasia and mucus production. The observed effect of ICG-001 on mucus production is in line with a trend toward a decrease in HDM-induced *Muc5ac* expression with ICG-001 treatment, which may have failed to reach statistical significance due to the high variation between mice. The RNA analysis of lung tissue exhibits greater within-group variance than AB/PAS staining, as the former is dependent on the relative proportion of RNA coming from the airway epithelial cells in the total lung tissue RNA pool. Furthermore, the design of our study may explain the discrepancy between the effect of ICG-001 on HDM-induced *Muc5ac* expression and production. We treated mice with ICG-001 after the initial HDM sensitization and we cannot be sure that the changes in *Muc5ac* gene expression were already induced during the initial sensitization phase. A sham-sensitized and HDM-treated control may be used in the future studies to obtain clarity on the potential of ICG-001 to suppress earlier HDM-induced changes during the sensitization phase, e.g., in *Muc5ac* gene expression. Furthermore, mRNA expression is usually transient and depends on the time point of measurement and the protein levels of mucins quantified by the staining can be considered as the most important measurement for goblet cell metaplasia. Our findings are in line with a previous study in which ICG-001 was shown to inhibit goblet cell metaplasia in a toluene diisocyanate treated mouse model of asthma (Yao et al., 2017).

It has been recently shown that ICG-001 prevented the differentiation of human airway epithelial progenitor cells into mucous-secreting cells and decreased *MUC5B* gene expression during culture at the air-liquid interface (Malleske et al., 2018). However, in addition to the lack of effect on *Muc5ac*, ICG-001 treatment did not affect *Muc5b* expression in HDM-treated mice. Further investigations into the molecular mechanisms responsible for the inhibition mucus production and goblet cell hyperplasia by ICG-01 will be addressed in future studies. The discrepancy in the effects of ICG-001 on *MUC5B* expression between our current *in vivo* observations and the findings *in vitro* (Malleske et al., 2018), where ICG-001 inhibited *MUC5B* expression could be due to differences in the cellular composition of airway epithelium between human and mice. Lineage tracing studies revealed that IL-13-induced goblet cells were derived from *FOXJ1*-expressing ciliated cells in human primary airway epithelial cells in ALI (Turner et al., 2011), but this was not demonstrated *in vivo* in an Ovalbumin-induced mouse model of allergic airway disease (Pardo-Saganta et al., 2013). Newly differentiated goblet cells generated in response to allergen in mice may originate predominantly from club cells present in the pseudostratified layer of airway epithelium (Park et al., 2007). Different types of progenitors for goblet cells may not be equally sensitive to the inhibitor. In addition to *Muc5ac*, ICG-001 treatment also had no effect on the gene expression of *Foxa2*, which was previously identified to be regulated by the β-catenin pathway (Mucenski et al., 2005). Of note, *Foxa2* is a transcriptional activator of E-cadherin (Song et al., 2010; Zhang et al., 2015), and the HDM-induced downregulation of *Foxa2* may have resulted in reduced expression of E-cadherin. In line with the lack of effect on *Foxa2*, we did not observe an effect of ICG-001 on E-cadherin expression. ICG-001 was able to significantly decrease the HDM-induced infiltration of macrophages, suggesting the important role of β-catenin/CBP signaling in HDM-induced pro-inflammatory responses in mice. Indeed, we have previously shown that ICG-001 inhibits the HDM-induced granulocyte macrophage-colony stimulating factor (GM-CSF) (Kuchibhotla et al., 2020), a cytokine that stimulates the production of macrophages. Alternatively, ICG-001 was not able to prevent the infiltration of eosinophils, neutrophils, and lymphocytes. This is in contrast to our *in vitro* data where we showed that ICG-001 reduced HDM-induced CCL20 levels in primary bronchial epithelial cells (Kuchibhotla et al., 2020), as neutrophils and T cells (predominantly Th17) are known to express CCL20 receptor CRR6. Moreover, ICG-001 was previously shown to be able to significantly decrease eosinophil and neutrophil influx into the airways induced by toluene diisocyanate in mice (Yao et al., 2017). These contrasting findings could be due to the difference in the mechanism of action of HDM and toluene diisocyanate. HDM activates pathogen recognition receptors (PRRs) like dectin-1 and Toll-like receptor (TLR)-4 resulting in the release of pro-inflammatory cytokines like CCL17, CCL20, IL-5, and IL-13 (Gregory and Lloyd, 2011; Post et al., 2014), whereas toluene diisocyanate acts on transient receptor potential melastatin 8 (TRPM8) resulting in the release of IL-25, IL-4, and IL-13 (Kim et al., 2017a). This suggests that TRPM8, but not PRR activation is regulated by β-catenin/CBP signaling. As HDM is a major allergen responsible for the airway inflammation in Type-2 driven, atopic asthma, our model may be more relevant for better understanding of the role of β-catenin/CBP signaling in allergic airway inflammation.

Here, we show that ICG-001 attenuates HDM-induced goblet cell metaplasia independent of HDM-induced airway inflammation. Inhibition of β-catenin/CBP pathway could be an alternative strategy to regulate mucus hypersecretion in asthma. Future studies should be directed toward more targeted delivery of the ICG-001 to specific airway epithelial cell types for increased efficiency of the drug.

## Data Availability Statement

The raw data supporting the conclusions of this article will be made available by the authors, without undue reservation.

## Ethics Statement

The animal study was reviewed and approved by University of Newcastle Animal Care and Ethics Committee.

## Author Contributions

VK was involved in formal analysis, project administration, validation, and writing the original draft of the manuscript. MS and AR were involved in conceptualization, methodology, investigation, formal analysis, project administration, and validation of the project. IH and MN were involved in supervision of the project. PH and DK were involved in conceptualization, funding acquisition, and supervision of the project. All authors have contributed significantly to the manuscript, read the manuscript, agreed with its content, and approve the submission of this manuscript.

## Conflict of Interest

The authors declare that the research was conducted in the absence of any commercial or financial relationships that could be construed as a potential conflict of interest.

## Publisher’s Note

All claims expressed in this article are solely those of the authors and do not necessarily represent those of their affiliated organizations, or those of the publisher, the editors and the reviewers. Any product that may be evaluated in this article, or claim that may be made by its manufacturer, is not guaranteed or endorsed by the publisher.
