# Supplementary material for: Inhibition of β-Catenin/CREB Binding Protein Signaling Attenuates House Dust Mite-Induced Goblet Cell Metaplasia in Mice
Source: Front Physiol. 2021 Jul 27;12:690531. doi: 10.3389/fphys.2021.690531 (PMC8353457; doi:10.3389/fphys.2021.690531)
Supplement: Supplementary file 1 [file Image_1.pdf]

Inhibition of β-catenin/CREB binding protein signaling attenuates house dust mite-induced goblet cell metaplasia in mice

Virinchi N. S. Kuchibhotla[1,2,3,4]*, Malcolm R. Starkey[2,5,6]*, Andrew T. Reid[1,7], Irene H. Heijink[3,4,8], Martijn C. Nawijn[3,4], Philip M. Hansbro[1,2,9]*, Darryl A. Knight[1,2,10,11]*

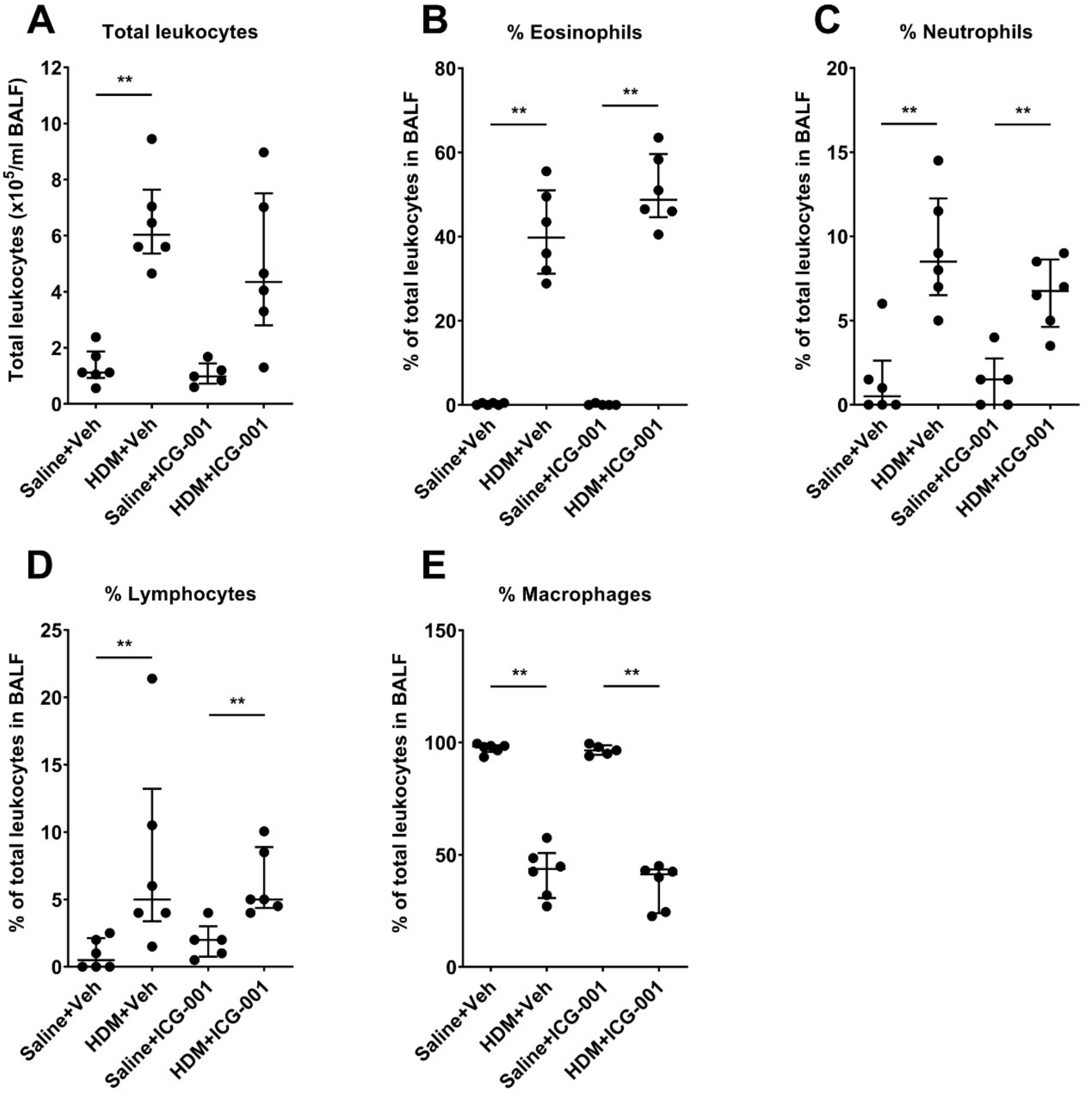

Figure S1: ***ICG-001 does not influence HDM-induced airway inflammation and AHR.*** Numbers of **(A)** total leukocytes (n=5-6), and percentage of **(B)** eosinophils (n=5-6), **(C)** neutrophils (n=5-6), **(D)** lymphocytes (n=5-6), and **(E)** macrophages (n=5-6) present in BAL fluid of mice. Data is presented as median ± IQR; n=5-6, **p<0.01, Mann–Whitney U test.
